# A multi-stimuli responsive switch as a fluorescent molecular analogue of transistors†
†Electronic supplementary information (ESI) available: Detailed experimental procedures and additional data on the characterization of **1**. See DOI: 10.1039/c5sc03395k



**DOI:** 10.1039/c5sc03395k

**Published:** 2015-11-19

**Authors:** Iluminada Gallardo, Gonzalo Guirado, Jordi Hernando, Sandy Morais, Gemma Prats

**Affiliations:** a Departament de Química , Universitat Autònoma de Barcelona , 08193 Cerdanyola del Vallès , Spain . Email: Gonzalo.Guirado@uab.cat ; Email: Jordi.Hernando@uab.cat

## Abstract

Although the quantum nature of molecules makes them specially suitable for mimicking the operation of digital electronic elements, molecular compounds can also be envisioned to emulate the behavior of analog devices. In this work we report a novel fluorescent three-state switch capable of reproducing the analog response of transistors, an ubiquitous device in modern electronics. Exploiting the redox and thermal sensitivity of this compound, the amplitude of its fluorescence emission can be continuously modulated, in a similar way as the output current in a transistor is amplified by the gate-to-source voltage.

## Abstract 2

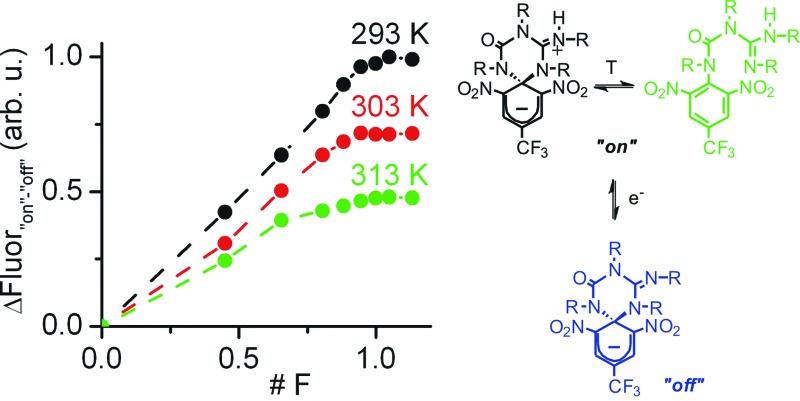
A redox-interconverting molecular switch is reported that enables continuous thermal amplification of its fluorescence, thus mimicking the response of transistors.

## Introduction

In the past years, much effort has been devoted to the development of molecular systems that mimic the behavior of digital electronic elements used for data and signal processing.^1,2^ A successful strategy towards this goal relies on the design of multistate compounds responding to external stimuli, which has enabled the preparation of a wealth of molecular analogues of these elements ranging from simple binary switches to complex logic gates and devices.^1b,2b,3–8^ All these examples benefit from the quantum character of molecules, which can only exist in certain defined states and, therefore, act as the discrete valued elements required in digital electronics. But though inherently digital in nature, stimuli-responsive molecular systems can also be envisioned to emulate the functioning of analog electronic devices, which present continuously variable instead of discrete output signals.^9^

In all probability, the most relevant analog devices are transistors, three-electrode circuit elements applied for signal switching and amplification in modern electronics. In spite of this, little attention has so far been paid to the development of molecular switching compounds aiming at reproducing their analog response.^8^ To date this has been mainly achieved by covalently tethering an organic emitter to several photochromic units, which can be reversibly photoisomerized between fluorescence quenching and nonquenching states. Variation of the light excitation intensities used to simultaneously trigger the forward and backward photochromic reactions allows controlling the concentration ratio of these states and, as such, all-photonic continuous modulation of the total emission registered.^10–12^ More recently, similar fluorescent constructs have been prepared using ionic quenching receptors, with which the analog operation of triode vacuum-tubes, the precursors of transistors, has been mimicked in solution using chemical signals.^13^

In this work we report a novel strategy to accomplish analog transistor-like behavior with stimuli-responsive compounds, which exploits the combined use of electrical and thermal inputs. A major advantage is expected from this approach, since signal modulation is to be achieved for the first time through variation of an intensive property of the system (*i.e.* temperature). As a result, this should allow removing the need to adjust the stimulus amplitude (*i.e.* excitation intensity^10–12^ or ion concentration^13^) to the population of active molecules in the sample when targeting defined output levels.

## Results and discussion

### Design and synthesis of the multi-stimuli responsive switch

**Scheme 1:**
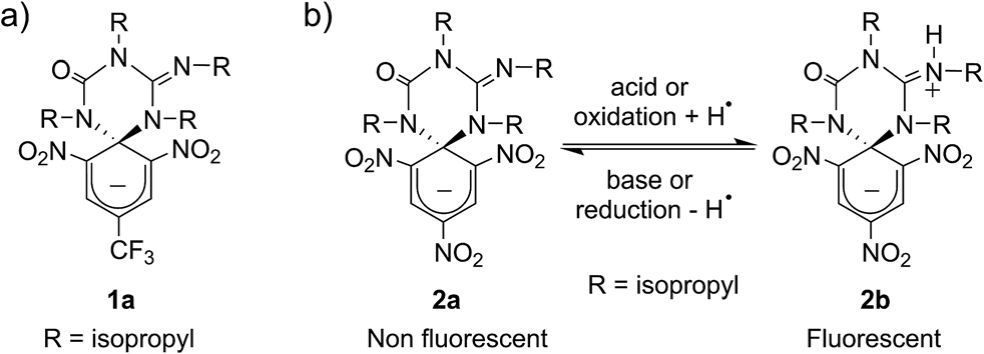
(a) Structure of multi-stimuli responsive switch **1**. (b) Chemical and electrochemical interconversion of switch **2** between its anionic and zwitterionic states. In the redox transformation of **2a** into **2b**, the solvent (*e.g.* acetonitrile) is used as the source of hydrogen atoms.

Scheme 1a shows the structure of the anionic state of dyad **1**, the compound developed in this study to display transistor-like behavior. Inspired by our previous work on a similar switch (**2** in Scheme 1b),^14–16^ **1** was designed to present a spirocyclic structure comprising two different units, a cyclohexadiene fluorophore and a cyclic triazene moiety responding to external stimuli. Of particular interest is the guanidine group of this moiety, which was shown to allow reversible interconversion between the anionic and zwitterionic states of **2** *via* protonation and deprotonation processes induced by: (i) acid–base addition, or (ii) oxidation and reduction followed by hydrogen atom abstraction from the solvent and elimination, respectively.^15,16^ Since cyclohexadiene emission was selectively quenched by the deprotonated state of the guanidine group, this made **2** behave as a fluorescent switch driven by chemical and electrochemical stimuli.^16^ To broaden the sensitivity of this system to additional external inputs for mimicking the analog response of transistors, compound **1** was derived by replacing one of the nitro groups of **2** with a trifluoromethyl moiety. This should lower the substituent effect on the resonance stabilization of the anionic cyclohexadiene fragment,^17^ thus decreasing the overall thermal stability of the spirocyclic structure of the dyad and, as such, allowing further modulation of its fluorescent behavior by means of temperature variations.

**Scheme 2:**
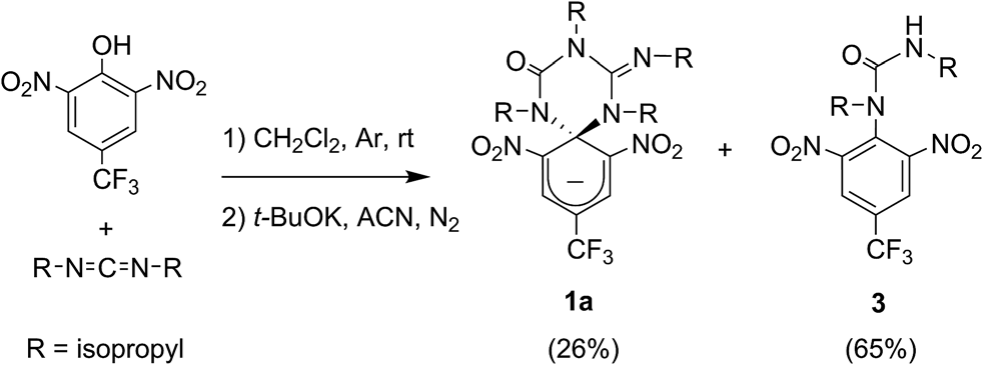
Synthesis of the anionic state of the switch **1a**.

**Fig. 1:**
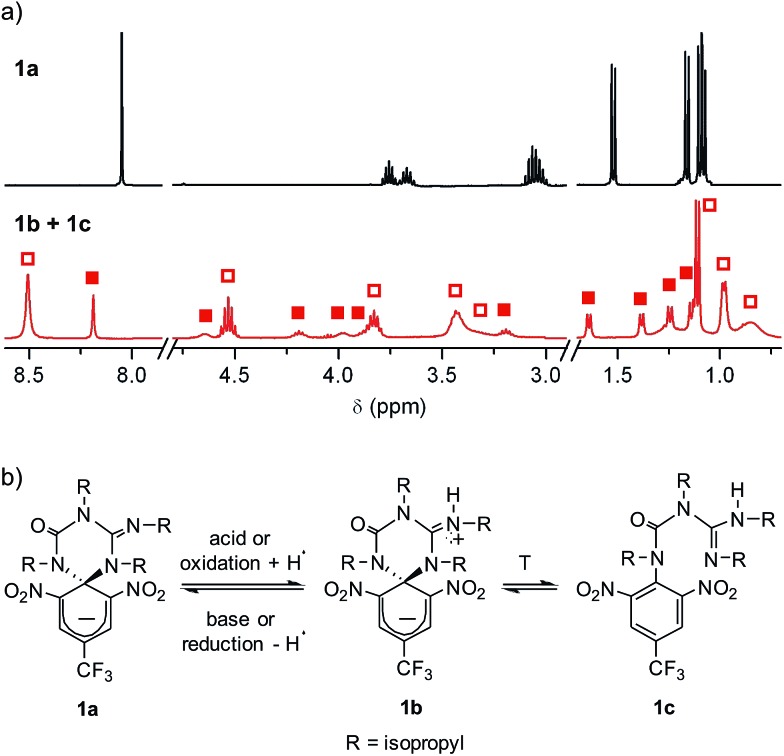
(a) ^1^H NMR (CD_3_CN, 400 MHz) spectra at 298 K of **1a** and the equilibrium mixture of **1b** and **1c**. Solid and hollow squares are used to discriminate the signals corresponding to **1b** and **1c**, respectively. For the sake of clarity, the intensity of the signals at *δ* > 3.0 ppm has been magnified (×3). (b) Interconversion between **1a** and the equilibrium mixture of **1b** and **1c**. In the redox conversion of **1a** into **1b** + **1c**, acetonitrile is used as the source of hydrogen atoms.^15^

Compound **1** was directly synthesized by reaction between 2,6-dinitro-4-(trifluoromethyl)phenol and *N*,*N*′-diisopropylcarbodiimide (Scheme 2). After removal of the aromatic by-product **3** formed in this reaction, an equimolar amount of base was added to yield the anionic state of the target compound (**1a**). In analogy to previously described switch **2**,^16^ acid addition to organic solutions of **1a** resulted in selective protonation of its guanidine group. In this case, however, the formation of a mixture of two different products was observed, as clearly demonstrated by ^1^H NMR (Fig. 1a). For the deprotonated product **1a**, only one set of signals was observed at 298 K in CD_3_CN, consisting of a singlet at *δ* = 8.05 ppm for the two cyclohexadiene protons, four different multiplets at *δ* = 3.80–2.98 ppm for the –CH– isopropyl nuclei, and four methyl doublets at *δ* = 1.53–1.07 ppm. Upon protonation, these signals disappeared and split into two new groups. Thus, two new uncorrelated singlets were found at *δ* = 8.19 and 8.51 ppm, while several multiplets were registered in the aliphatic region suggesting the coexistence of more than one protonated species (Fig. 1a). In spite of this, a single value of molecular mass was determined by HR-MS for this mixture, which was chromatographically irresoluble and reverted back quantitatively to **1a** after base addition. These results and the additional measurements conducted (see below) suggested that protonation of **1a** afforded a mixture of two interconverting isomers, which we assigned to the zwitterionic state of **1** (**1b**) and the neutral aromatic compound **1c** resulting from the ring-opening of its spiro-cyclic structure (Fig. 1b). This proved the success of our molecular design, which aimed to reduce the thermal stability of the bicyclic scaffold of **1** by introducing a trifluoromethyl substituent. In this way, we converted the two-state switch **2** into a three-state multi-stimuli responsive system.

### Optical and electrochemical properties of **1**

**Fig. 2:**
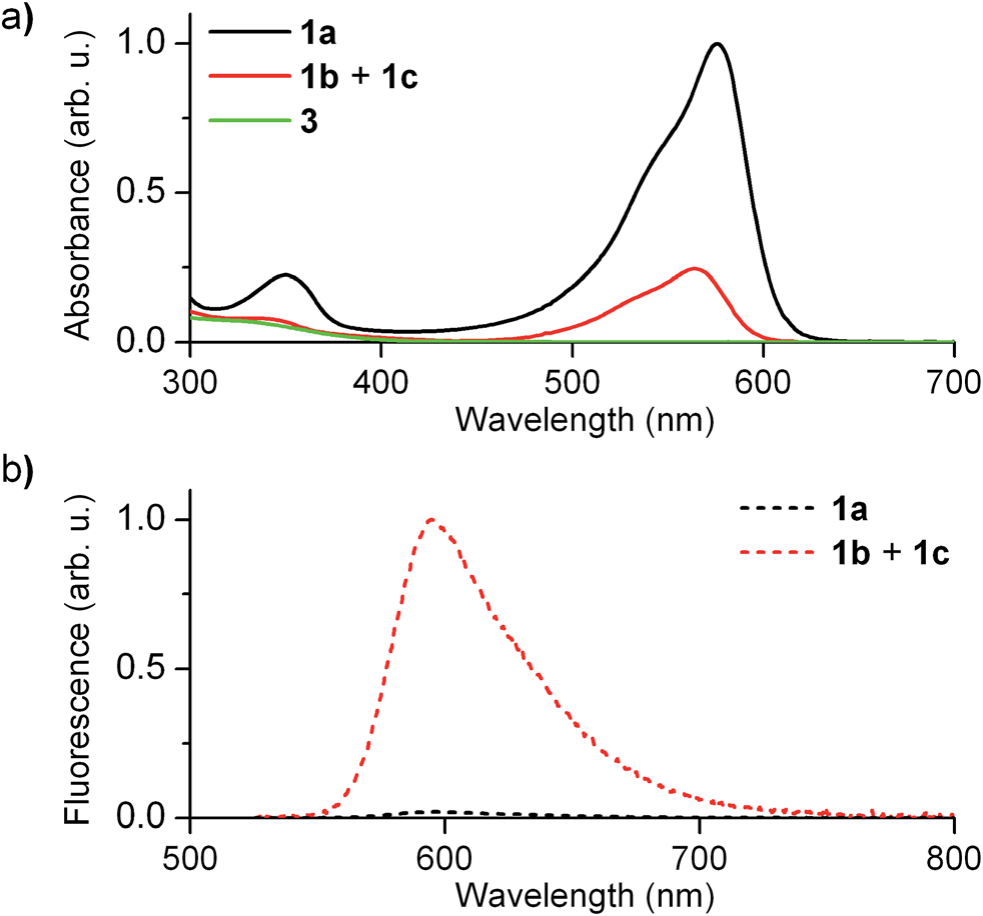
(a) Absorption and (b) fluorescence (*λ*_exc_ = 532 nm) spectra of **1a** (1.0 × 10^–5^ M) and the equilibrium mixture of **1b** and **1c** (1.0 × 10^–5^ M) in acetonitrile at 298 K. In (a) the absorption spectrum of **3**, the by-product formed in the synthesis of **1**, is also shown.

Fig. 2 shows the absorption and fluorescence spectra measured for the different states of **1** at 298 K. Pure **1a** preserved the main features previously described for **2a**.^15,16^ Thus, it showed strong visible light absorption arising from its cyclohexadiene chromophore (*ε* **_1a_** = 14 100 cm^–1^ M^–1^ at 576 nm), which bathochromically shifted due to the introduction of the CF_3_ group (*λ*_abs,max_ = 576 and 526 nm for **1a** and **2a** in acetonitrile, respectively). In addition, very dim fluorescence emission was registered for **1a** (*Φ*_fl,_ **_1a_** = 0.01 in acetonitrile), which can be ascribed to chromophore quenching *via* photoinduced electron transfer from the deprotonated guanidine moiety.

Upon acid-induced conversion of **1a** into the mixture of **1b** and **1c**, clear changes in these optical properties were observed. First, a large decrease in absorption was measured, although minimal changes in the shape of the spectrum were detected (*λ*_abs,max_ = 564 nm for **1b** + **1c**, Fig. 2a). Taking into account that **3**, an aromatic compound analogous to **1c**, only absorbs in the UV-violet region (*λ*_abs_ < 400 nm, Fig. 2a), we concluded that: (i) the visible absorption band at *λ*_abs_ ∼ 575 nm of the neutral state of the system can be fully assigned to **1b**, which actually presents the same cyclohexadiene chromophore as **1a**; (ii) the decrease of this signal upon protonation should be attributed to the low thermal stability of **1b** and its partial conversion into non absorbing **1c**. Indeed, the ∼3-fold absorption decrement observed in Fig. 2a is in agreement with the 1 : 3.3 molar ratio found by ^1^H NMR for the **1b** : **1c** equilibrium mixture in acetonitrile at 298 K. In spite of this, a large increase in emission intensity (∼25-fold) was observed for this mixture at *λ*_exc_ = 532 nm (*i.e.* when selectively exciting **1b**). As expected, this indicates that the quenching effect of the guanidine group on cyclohexadiene chromophore emission is suppressed upon protonation, thus producing a highly fluorescent zwitterionic species (*Φ*_fl,_ **_1b_** = 0.76 in acetonitrile).

**Fig. 3:**
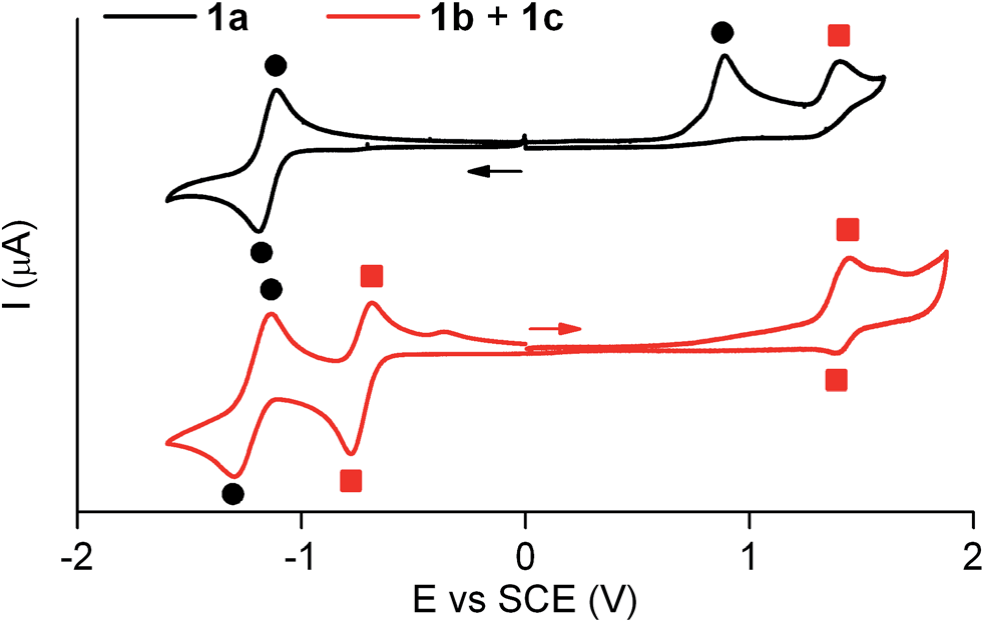
Cyclic voltammograms of **1a** (4.6 × 10^–3^ M) and the equilibrium mixture of **1b** and **1c** (4.9 × 10^–3^ M) in acetonitrile + 0.1 M *n*-Bu_4_NPF_6_ at 298 K (scan rate: 0.5 V s^–1^). Solid circles and squares are used to assign the electrochemical waves arising from **1a** and **1b** + **1c**, respectively. Arrows indicate the direction of the potential scan in each case.

Similar electrochemical properties were found for **1** with respect to the previously studied switch **2**.^15^ Fig. 3 shows the cyclic voltammogram of **1a** in acetonitrile at 298 K. In the first cathodic scan, a one-electron reversible reduction wave at *E* ^0^ = –1.14 V (*vs.* SCE) was found, which can be attributed to the reversible formation of the corresponding dianion. In the subsequent anionic counter scan, two oxidation peaks were observed: a one-electron irreversible oxidation peak at *E*_pa_ = +0.89 V (*vs.* SCE) and a one-electron reversible peak at *E* ^0^ = +1.37 V (*vs.* SCE). Since the former indicated the evolution of the oxidized neutral radical of **1a** into a new product, a controlled potential electrolysis of **1a** at +1.00 V (*vs.* SCE) was conducted. Characterization of the product formed by ^1^H NMR, cyclic voltammetry and UV-vis absorption and fluorescence spectroscopies demonstrated quantitative transformation of **1a** into a **1b** + **1c** mixture after the passage of 1 F. According to our electrochemical experiments and the well-known hydrogen atom donor properties of acetonitrile,^15,18^ this transformation should proceed *via* one-electron oxidation of **1a** followed by hydrogen atom abstraction from the solvent with an estimated rate constant of 10^6^ s^–1^ (see Scheme S1 in ESI†).

The cyclic voltammogram of the **1b** + **1c** equilibrium mixture in acetonitrile at 298 K is also depicted in Fig. 3, where a one-electron reversible oxidation wave is seen at *E* ^0^ = +1.37 V (*vs.* SCE) in the anodic scan. This wave matches that previously found for **1a** upon irreversible oxidation, which confirms that it arises from **1b** + **1c**. In the cathodic counter scan, a first one-electron pseudo-reversible peak *E*_pc_ = –0.78 V (*vs.* SCE) was detected, which suggested irreversible reduction-induced transformation of **1b** + **1c**. As such, the **1b** + **1c** mixture was subjected to a controlled potential electrolysis at –1.00 V (*vs.* SCE), which led to the quantitative formation of **1a** after the passage of 1 F as demonstrated by ^1^H NMR, cyclic voltammetry and UV-vis absorption and fluorescence spectroscopies. In this case, the electrochemically-induced conversion of **1b** + **1c** into **1a** is proposed to take place *via* one-electron reduction of the reactants and subsequent elimination of a hydrogen atom (*k* = 78 s^–1^, see Scheme S1 in ESI†), which is expected to eventually evolve into molecular H_2_.^19^

### Fluorescence switching of **1**

**Fig. 4:**
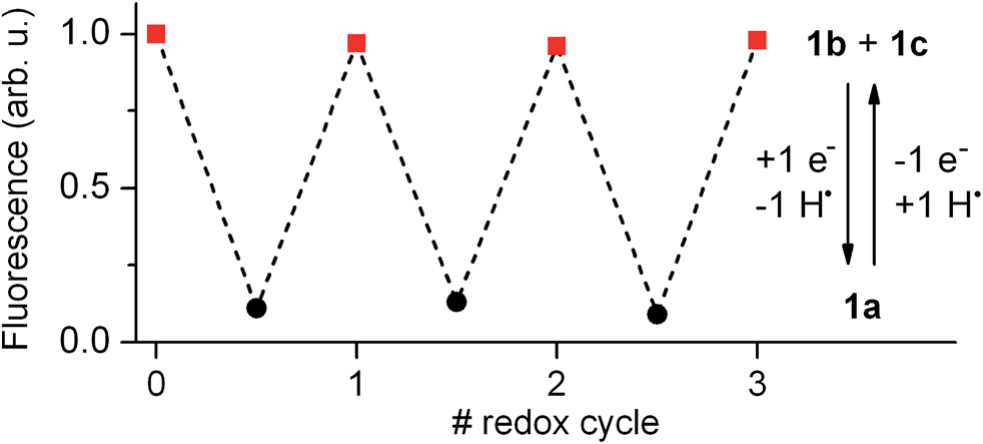
Fluorescence intensity of **1** in acetonitrile at 298 K (5.0 × 10^–3^ M + 0.1 M *n*-Bu_4_NPF_6_) upon consecutive exhaustive electrolysis (1 F, 2.41 C) at *E*_ap_ = –1.00 V and +1.00 V (*vs.* SCE) to induce reversible interconversion between **1b** + **1c** and **1a** *via* reduction and oxidation processes, respectively. In the redox conversion of **1a** into **1b** + **1c**, the solvent (*e.g.* acetonitrile) is used as the source of hydrogen atoms.

Our electrochemical experiments revealed the capability of **1** to switch between its anionic and neutral states electrochemically, which present different fluorescent properties. To demonstrate the bidirectional redox fluorescence switching of this compound, sequential reductive and oxidative exhaustive electrolysis were applied and the interconversion processes induced were monitored *via* emission measurements. As shown in Fig. 4, robust and reproducible fluorescence modulation was observed by reversible electrochemical transformation between **1a** and **1b** + **1c**, a situation that could also be reproduced by means of consecutive additions of acid and base (see Fig. S1 in ESI†). Therefore, **1** behaves as a pH- and redox-sensitive fluorescent switch by interconversion between its anionic and neutral states, thus reproducing the behavior of **2** despite the low thermal stability of the zwitterionic species **1b**.

Nevertheless, **1** presents an additional switching feature with respect to **2**, which is crucial to emulate the analog behavior of transistors: the amplitude of the fluorescence modulation between the “off” (**1a**) and “on” states (**1b** + **1c**) of the system ultimately depends on the **1b** : **1c** concentration ratio upon acid- or redox-induced protonation, since only the former of these species is emissive. As such, increasing the molar fraction of **1b** in the mixture should allow continuous amplification of the fluorescence response of the switch.

**Fig. 5:**
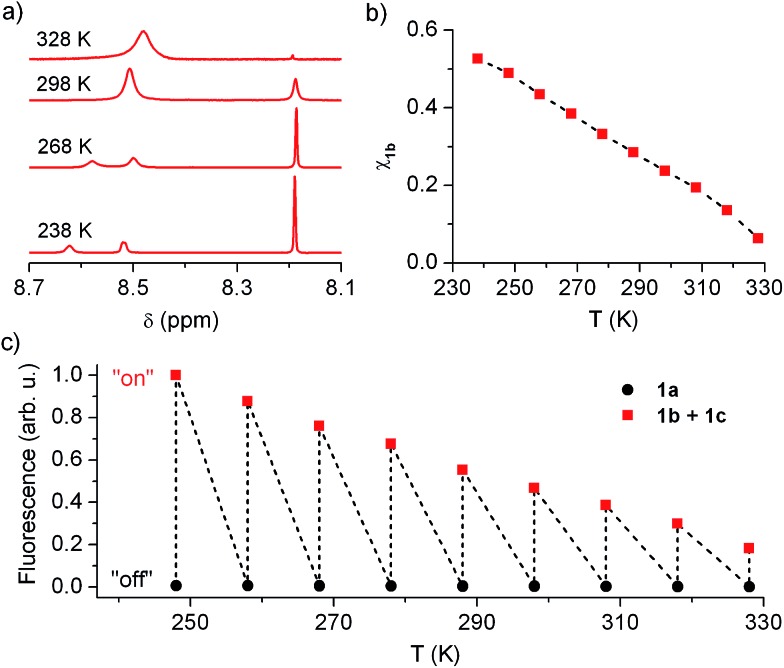
(a) Low field region (*δ* ∼ 8.7–8.1 ppm) of the ^1^H NMR (CD_3_CN, 250 MHz) spectrum of the neutral state of **1** at variable temperatures. (b) Temperature dependence of the molar fraction of **1b** in the neutral state of **1** in acetonitrile solution from NMR data. (c) Temperature dependence of the fluorescence emitted by acetonitrile solutions of **1a** and **1b** + **1c** (1.0 × 10^–5^ M).

In view of this, we investigated whether the composition of the neutral state of **1** could be modulated externally and, in a first step, we considered thermal control. Fig. 5a plots the low field region of the ^1^H NMR spectrum of the equilibrium mixture **1b** + **1c** registered at different temperatures in acetonitrile (see Fig. S2 in ESI† for the complete spectra), where the signals for the cyclohexadiene protons of **1b** (*δ* ∼ 8.19 ppm) and the aromatic protons of **1c** (*δ* ∼ 8.50–8.60 ppm) are found. Two different dynamic effects were observed for those signals at distinct thermal ranges. First, the **1c** signal broadened and ultimately split into two different peaks upon cooling below room temperature. Since no concomitant changes were observed for the **1b** signal at *T* < 288 K, we ascribe this behavior to the hindered rotation of the bulky urea substituent around the aromatic moiety of **1c**, which must be sufficiently slowed down at low temperatures as to be resolved by NMR. By contrast, the **1b** and **1c** tautomerization process must occur at much lower rates at such conditions, the **1b** signal thus preserving its lineshape and frequency (*i.e. k* **_1b_**_→_ **_1c_** and *k* **_1c_**_→_ **_1b_** must be lower than 1 s^–1^ at *T* < 288 K).^20^ However, broadening of both **1b** and **1c** signals was observed when heating above 288 K, which demonstrates acceleration of the **1b**–**1c** interconversion reaction with temperature. Actually, lineshape analysis of those signals allowed the tautomerization rate constants to be estimated,^20^ which increased from *k* **_1b_**_→_ **_1c_** = 1.16 s^–1^ to *k* **_1b_**_→_ **_1c_** = 196 s^–1^ within the 288–328 K interval (see Table S1 in ESI†).

More importantly, the composition of the **1b**–**1c** equilibrium mixture and, therefore, the equilibrium constant of the tautomerization process (*K*_eq,_ **_1b_**_,_ **_1c_**) could be determined at each temperature from the integrals of the NMR signals (see Table S1 in ESI†). In this way we could demonstrate that the stability of the spirocyclic structure of the protonated state of **1** dramatically decreased with temperature. In particular, **1b** relative concentration in the neutral state of the switch could be tuned from 53% at 238 K down to 6% at 328 K (Fig. 5b), a behavior that was found to be fully reversible upon a complete warming–cooling cycle (see Fig. S3 in ESI†) and further confirmed by absorption measurements (see Fig. S4 in ESI†).

As expected, thermal variation of **1b** concentration in the neutral state of the switch enabled amplitude modulation of its fluorescence response. This is clearly proven by Fig. 5c, where the temperature dependences of the emission arising from acetonitrile solutions of **1a** and **1b** + **1c** are plotted. While negligible thermally induced changes were observed for the “off” state of the system, a continuous variation of the “on” state fluorescence was found with temperature. In this way, the “on”–“off” emission contrast of the switch could be amplified up to 455% when cooling down the sample from 328 K to 248 K, the largest temperature range available for our optical experiments. An even larger amplification effect was predicted from pure concentration arguments, since **1b** molar fraction increased around 680% along the 328–248 K interval according to NMR data. Most probably, variation of **1b** absorptivity and *Φ*_fl_ values with temperature accounted for the ∼1.5-fold lower emission changes monitored, which are however large enough as to permit significant amplitude switching of **1** using not only chemical or electrochemical stimuli but also temperature variations.

### Mimicking the analog behavior of transistors with **1**

Although a wealth of fluorescent molecular compounds and materials have been developed whose emission properties can be modulated using acid–base,^21^ redox^22^ or thermal^23^ inputs, the number of systems reported to simultaneously respond to more than one of those stimuli is still rather limited.^24–26^ Indeed, to the best of our knowledge, no fluorescent switch capable to function upon application of combined acid–base, electrochemical and thermal stimuli has been described so far. This makes **1** a rather unique case of multiresponsive compound, a type of systems of particular interest for the preparation of multifunctional molecular devices and materials, such as complex logic gates^2b,8^ and multimode data storage media with increased information density.^6,27^ As a proof of this, we exploited herein the electrochemical and thermal sensitivity of **1** for mimicking the analog behavior of transistors.

**Fig. 6:**
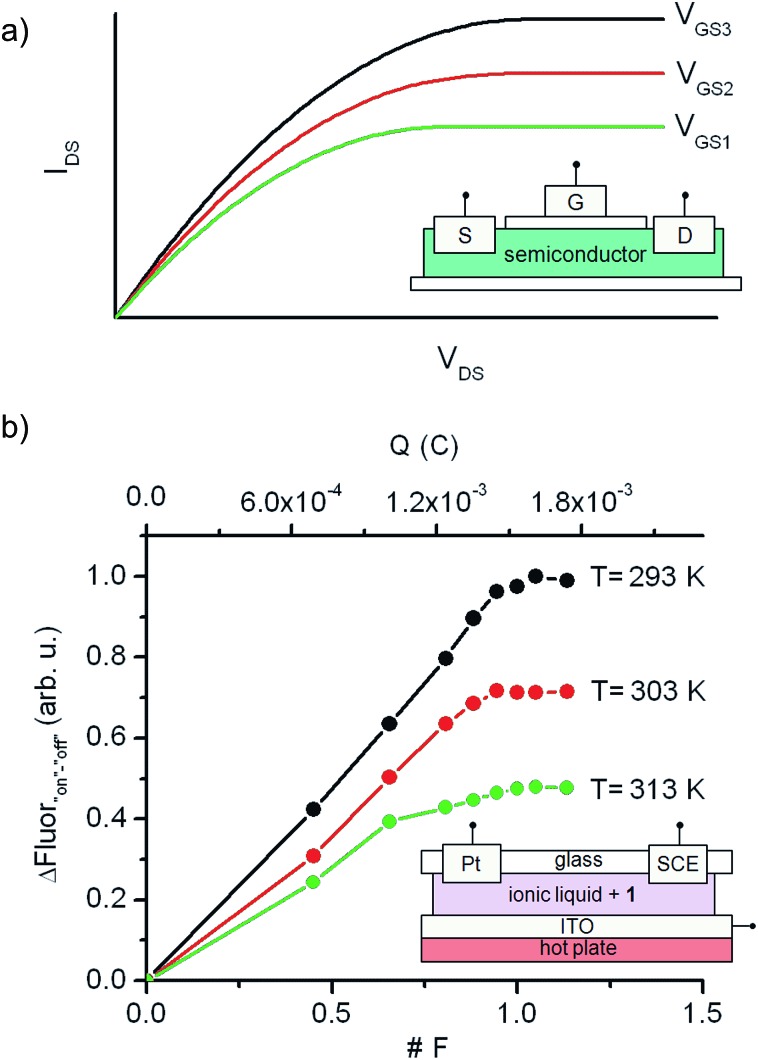
(a) Typical current–voltage behavior of a FET, where the source-to-drain current (*I*_DS_) at a given voltage (*V*_DS_) can be amplified upon application of a gate-to-source electric field (*V*_GS_). The inset shows a schematic representation of the transistor structure. (b) Analog transistor-like response of an ionic liquid thin film of **1** (2 × 10^–5^ M) connected to working (ITO), auxiliary (Pt) and reference (SCE) electrodes and placed onto a hot plate. The temperature-tunable I/O curves were obtained by plotting the fluorescence amplitude measured when interconverting from the “off” to the “on” state of the switch *via* oxidative electrolysis (*E*_ap_ = +1.30 V (*vs.* SCE)). Both the absolute (in C) and relative (in F) amount of charges injected in these experiments are given in the plot.

Fig. 6a depicts the principal components and typical response of field-effect transistors (FET), the most common type of transistor in current electronic circuits. It consists of a semiconductor connected to a three-electrode system (source (S), drain (D) and gate (G)), where the current flowing from the source to the drain (output signal, *I*_DS_) does not only depend on the voltage applied between them (input signal, *V*_DS_), but can also be tuned upon application of a variable gate-to-source electric field (*V*_GS_). In particular, *V*_GS_ controls the shape and size of the conductive channel in the semiconductor through which current flows from the source to the drain, thus allowing the output signal of the device to be amplified (by enlarging the channel) or switched off (by squeezing it).

To mimic this behavior, a conductive and nonvolatile thin film of the ionic liquid 1-ethyl-3-methylimidazolium bis(trifluorosulfonyl)imide ([EMIM][NTf2]) was doped with a 2 × 10^–5^ M content in **1a**, sandwiched between a glass substrate and an ITO layer, put in contact with two additional auxiliary (Pt) and reference electrodes (SCE), and finally placed onto a hot plate (Fig. 6b). In such a construct, the analog functioning of FETs was devised to be emulated by: (i) measuring the fluorescence of the film (output signal) when electrochemically interconverting between the emissive and nonemissive states of **1** upon controlled electrolysis at defined potentials (input signal); (ii) modulating the resulting input/output (I/O) response through temperature variations, which must function as the gate-to-source voltages applied in field-effect transistors. As a proof of concept, Fig. 6b shows for the results obtained in this way at three different temperatures (293, 303 and 313 K), for which the “on”–“off” fluorescence amplitudes measured are plotted against the charge injected (*Q*) during the oxidative electrolysis of **1a**. It must be noted that *Q* is a time-dependent parameter, in contrast to the *V*_DS_ variable used when recording the I/O curves of FETs. However, *Q* is also related to the electronic properties of the switching system and, more importantly, to the interconversion between its different states, thus emerging as an appropriate variable to monitor the transistor-like behavior of **1**.

At *Q* = 0, minimal fluorescence was measured at the three temperatures of choice, since the molecular switch remained in its nonemissive **1a** state. A nearly linear increase of the emission with the charge injected was then registered upon oxidative electrolysis, which led to **1a** → **1b** + **1c** conversion *via* radical formation and hydrogen atom abstraction from the ionic liquid solvent. This behavior resembles that observed in the linear region of the I/O curves of FETs at low *V*_DS_ values. However, as approaching to passage of 1 F and, therefore, quantitative transformation of **1a** into **1b** + **1c** was achieved, the signal saturated and further injection of charge did not result in an additional rise in emission, thus mimicking the saturation of the transistor response at large *V*_DS_ values. Noticeably, the amplitude of the fluorescence curves measured in this way dramatically varied with temperature, as expected due to the thermal dependence of the equilibrium constant of the tautomerization process between **1b** and **1c**. In particular, a ∼100% increase in maximal fluorescence signal was determined when cooling down the system from 313 to 293 K, since stabilization of the spirocyclic structure of the emissive species **1b** takes place at lower temperatures. It is worth mentioning that this value is larger than that measured in acetonitrile solution for the 318–298 K range (∼60%), which we ascribe to the different thermal dependence of *K*_eq,_ **_1b_**_,_ **_1c_** with the solvent. Despite this, Fig. 6b clearly shows that similar I/O curves to those typically displayed by FETs can be obtained by exploiting the multi-stimuli responsive behavior of switch **1**, thus demonstrating the capability of this compound to behave as a molecular analogue of transistors.

When comparing the performance of our molecular system with that of transistors, it must be noted that it provides a different type of output signal as well as operates under distinct external stimuli, which hampers the direct use of our molecular switch as a substitute of transistors in current circuits. However, it enables its application in other fields where transistor-like responses could be exploited. Actually, fluorescent molecular analogues of electronic components are currently being successfully applied as local probes of relevant biological systems and processes, since they can report on physical and (bio)chemical conditions *in vivo*, with minimal perturbance, high sensitivity and sub-micrometer resolution.^4,28^ Of special interest in this area is the development of compounds capable of simultaneously detecting and quantifying several analytes, which should not only broaden the density of sensory information but also allow direct screening of medical conditions related to a combination of different biological indicators. Lab-on-a-molecule^24e,29^ and keypad lock systems^30^ are some of the most popular strategies to achieve this goal, and they have already been used to investigate complex processes such as enzymatic activity,^31^ neuronal exocytosis^32^ or mitochondrial metabolism.^33^ Among the variety of analytes that could be monitored by these molecular devices, the multiplexed sensing of pH,^34^ redox state^35^ and temperature,^36^ the input signals of compound **1**, would be of paramount importance, because these parameters are known to change during regular cellular processes^37^ and their abnormal values are often related to common diseases (*e.g.* cancer^38^). Therefore, taking advantage of the multi-stimuli responsive, transistor-like behavior of **1**, this and related compounds could be envisaged as lab-on-a-molecule systems for the simultaneous *in vivo* detection and mapping of cellular redox state and temperature (or pH and temperature).

## Conclusions

A novel molecular switch was developed in this work to mimic the analog operation of transistors, which consists of a spirocyclic dyad made of a cyclohexadiene fluorophore and a triazene group undergoing reversible redox and acid–base interconversion between quenching (“off”) and nonquenching (“on”) states. By selectively decreasing the stability of the spirocyclic structure of the “on” state, an equilibrium mixture of the fluorescent species and an optically-inactive tautomer is formed, whose composition is largely dependent on temperature. As a result, the amplitude of the “on”–“off” emission modulation of the switch can be thermally controlled, in a similar way as the output current in a field-effect transistor is amplified by the gate-to-source voltage. Such a combined thermal and chemical sensitivity of the fluorescence molecular response can be exploited for the design of lab-on-a-molecule systems enabling multiplexed sensing of cellular temperature, redox state and/or pH, some of the most relevant variables involved in regular biological processes and several common diseases.
