# Supplementary material for: A multi-stimuli responsive switch as a fluorescent molecular analogue of transistors
Source: Chem Sci. 2015 Nov 19;7(3):1819–25. doi: 10.1039/c5sc03395k (PMC5604542; doi:10.1039/c5sc03395k)
Supplement: Supplementary file 1 [file SC-007-C5SC03395K-s001.pdf]

Electronic Supplementary Material (ESI) for Chemical Science.
This journal is © The Royal Society of Chemistry 2015

Electronic Supplementary Information

A Multi-Stimuli Responsive Switch as a Fluorescent Molecular Analogue of Transistors

Iluminada Gallardo, Gonzalo Guirado*, Jordi Hernando,* Sandy Morais and Gemma Prats

Contents

I. SYNTHESIS

II. OPTICAL AND ELECTROCHEMICAL CHARACTERIZATION

III. REDOX, ACID-BASE AND THERMAL SWITCHING

IV. BIBLIOGRAPHY

I. SYNTHESIS

Materials and methods: Reagents and chemicals were used as commercially purchased and without further purification. Flash column chromatography was performed on silica gel 60 Å, particle size 35-70 μm. ^{1}H NMR spectra were recorded on a Bruker DPX360 (360 MHz) and a Bruker AV-III400 (400 MHz) spectrometers. ^{13}C NMR spectra were recorded on a Bruker AV-III400 (100 MHz) spectrometer with complete proton decoupling. Proton chemical shifts are reported in ppm (δ) ($CDCl_3$, δ 7.26 or CD_3CN, δ 1.94). Carbon chemical shifts are reported in ppm (δ) ($CDCl_3$, δ 77.2 or CD_3CN, δ 1.32). High resolution mass spectra (HRMS) were recorded on an ESI-QTOF Bruker Daltonics microTOF-Q spectrometer.

Synthesis of 1 and *N*-(2,6-dinitro-4-trifluoromethylphenyl)-*N*,*N'*-diisopropylurea (3): To a dichloromethane solution (35 mL) of commercially available 2,5-dinitro-4-(trifluoromethyl)phenol (2.5 g, 9.9 mmols), a previously prepared dichloromethane solution (20 mL) of commercial diisorpopylcarbodiimide (12.0 g, 95.0 mmols) was slowly added. This mixture was stirred at room temperature and under Ar atmosphere for 4 h. Afterwards, the solvent was evaporated *in vacuo* and the resulting solid was redissolved in hot methanol (120 mL). Water (80 mL) was then added to this solution and it was stored at 0º for 72 h. After this treatment, the sample separated into a liquid and a jelly-like phases, which were separated by filtration. The gelatinous precipitate was dissolved in dichloromethane and subjected to successive flash column chromatographies (ethyl acetate:hexane 2:3) to isolate by-product **3** (2.43 g, 65% yield) and an irresoluble mixture of **1b** and **1c** (1.30 g, 26% yield). This mixture (0.10 g, 0.20 mmols) was then dissolved in anhydrous acetonitrile under N_2 atmosphere and then treated with previously dried solid *tert*-BuOK until no fluorescence was observed for the solution under irradiation with UV light (365 nm). The excess of base was removed by filtration and the solvent of the filtrate was evaporated *in vacuo* to obtain the pure potassium salt of **1a** as a solid reddish powder (0.11 g, 99% yield).

Pure compounds **1a** and **3**, and the equilibrium mixture of the two interconverting tautomers **1b** and **1c** were characterized by ^{1}H NMR, ^{13}C NMR and HR-MS. The ^{1}H NMR signals of the two products in the mixture were assigned on the basis of their integrals and COSY and temperature dependent experiments.

1a: ^{1}H NMR (400 MHz, CD_3CN, 298 K): δ = 8.05 (s, 2H), 3.76 (m, 1H), 3.67 (m, 1H), 3.12-2.98 (m, 2H), 1.52 (d, ^{3}J(H,H)=6.7 Hz, 6H), 1.16 (d, ^{3}J(H,H)=6.6 Hz, 6H), 1.10 (d, ^{3}J(H,H)=6.7 Hz, 6H), 1.08 (d, ^{3}J(H,H)=6.7 Hz, 6H); ^{13}C NMR (100.6 MHz, CD_3CN, 298 K): δ = 152.5, 143.4, 131.0 (q, ^{3}J(H,F)=3.0 Hz), 129.7, 126.4 (q, ^{1}J(H,F)=267.4 Hz), 96.0 (q, ^{2}J(H,F)=35.2 Hz), 79.7, 54.7, 50.8, 50.7, 48.7, 25.6, 22.3, 20.5, 20.2; HR-MS (ESI-QTOF) calcd. for [$C_{21}H_{30}F_3N_6O_5$+H]: 504.2320; found: 504.2308.

1b+**1c**: ^{1}H NMR (400 MHz, CD_3CN, 298 K): δ = 8.51 (s, 2H, **1c**), 8.19 (s, 2H, **1b**), 4.64 (m, 1H, **1b**), 4.53 (sept, ^{3}J(H,H)=6.7 Hz, 1H, **1c**), 4.19 (sept, ^{3}J(H,H)=6.6 Hz, 1H, **1b**), 3.97 (m, 1H, **1b**), 3.93-3.78 (m, 1H, **1b**, 1H, **1c**), 3.55-3.25 (m, 3H, **1c**), 3.19 (sept, ^{3}J(H,H)=6.7 Hz, 1H, **1b**), 1.64 (d, ^{3}J(H,H)=7.1 Hz, 6H, **1b**), 1.38 (d, ^{3}J(H,H)=7.1 Hz, 6H, **1b**), 1.24 (d, ^{3}J(H,H)=7.1 Hz, 6H, **1b**), 1.14 (d, ^{3}J(H,H)=7.1 Hz, 6H, **1b**), 1.11 (d, ^{3}J(H,H)=7.1 Hz, 12H, **1c**), 0.98 (d, ^{3}J(H,H)=7.1 Hz, 6H, **1c**), 0.93-0.74 (m, 12H, **1c**); ^{13}C NMR (100.6 MHz, CD_3CN, 298 K): δ = 157.1, 154.1, 150.6, 149.0, 142.1, 134.2, 132.5, 129.9 (q, ^{2}J(H,F)=35.9 Hz), 127.6 (q, ^{3}J(H,F)=3.0 Hz), 124.7, 124.4 (q, ^{1}J(H,F)=265.6 Hz), 121.9 (q, ^{1}J(H,F)=273.6 Hz), 97.2 (q, ^{2}J(H,F)=34.5 Hz), 82.8, 57.7, 55.1, 54.2, 51.8, 51.7, 51.4, 46.9, 43.3, 24.3, 23.7, 22.1, 21.1, 20.9, 20.2, 19.6, 18.6; HR-MS (ESI-QTOF) calcd. for [$C_{21}H_{31}F_3N_6O_5$+H]: 505.2386; found: 505.2385.

3: ^{1}H NMR (360 MHz, CD_3CN, 298 K): δ = 8.46 (s, 2H), 4.96 (d, ^{3}J(H,H)=6.4 Hz, 1H), 4.30 (sept, ^{3}J(H,H)=6.8 Hz, 1H), 3.84 (m, 1H), 1.01 (d, ^{3}J(H,H)=6.8 Hz, 6H); ^{13}C NMR (62.5 MHz, CD_3CN, 298 K): 155.4, 151.7, 132.9 (q, ^{4}J(H,F)=1.0 Hz), 131.8 (q, ^{2}J(H,F)=35.3 Hz), 126.9 (q, ^{3}J(H,F)=3.7 Hz), 123.0 (q, ^{1}J(H,F)=270.8 Hz), 52.2, 43.7, 22.8, 21.5; HR-MS (ESI-QTOF) calcd. for [$C_{14}H_{17}F_3N_4O_5$+H]: 379.1229; found: 379.1231.

II. OPTICAL AND ELECTROCHEMICAL CHARACTERIZATION

Materials and methods: UV-Vis absorption spectra were recorded using a HP 8452A spectrophotometer (Agilent) with chemstation software. Fluorescence spectra were recorded by means of a custom-made spectrofluorometer, where a cw diode laser (Z-laser, λ_{exc} = 532 nm) was used as excitation source and the emitted photons were detected in an Andor ICCD camera coupled to a spectrograph. In all cases spectroscopy quality solvents and 1-cm quartz cuvettes were used. Temperature was controlled using a refrigerated circulator bath (Huber MPC-K6) connected to the sample holder. Fluorescence quantum yields were determined for highly diluted solutions of the compounds of interest to prevent self-absorption processes (absorption < 0.05 at the excitation wavelength) and they were measured relative to *N,N'*-bis(butyl)-1,6,7,12-tetra-(4-*tert*-butylphenoxy)perylene-3,4:9,10-tetracarboxylic diimide in CH_2Cl_2 (Φ_f =1).[1]

Cyclic voltammograms were registered using a VSP100 BIOLOGIC potentiostat and a conical electrochemical cell equipped with an argon bubbling source for degassing, a glassy carbon working electrode (WE, d= 0.45 mm), a glassy carbon auxiliary electrode (CE, d= 3 mm) and a saturated calomel reference electrode (SCE, RE). All the potentials are reported versus a SCE isolated from the working electrode by a salt bridge. All measurements were performed in acetonitrile solution containing 0.1 M of *n*-Bu_4NPF_6 as supporting electrolyte. Electrolysis experiments at controlled potentials were undertaken with a EG&G Princeton Applied Research (PAR) 273A potentiostat and an electrochemical cell equipped with an argon bubbling source, a carbon graphite rod, an auxiliary platinum electrode and a SCE reference electrode. In these experiments both the reference and auxiliary electrodes were separated from the sample by a salt bridge. All electrochemical experiments were performed in acetonitrile solutions containing *n*-Bu_4NPF_6 (0.1 M) as supporting electrolyte. The products obtained were then characterized by ^{1}H NMR, cyclic voltammetry, UV-vis absorption spectroscopy and fluorescence spectroscopy.

III. REDOX, ACID-BASE AND THERMAL SWITCHING

Redox switching: To investigate the mechanism of the redox switching of **1**, cyclic voltammograms were measured at different scanning velocities (v, from 0.05 to 1.5 V/s) and the potential of the irreversible waves leading to **1a** → **1b**+**1c** and **1b**+**1c** → **1a** interconversions plotted vs log v. For both processes, linear dependences were observed with slopes close to 30 mV, which are consistent with these electrochemically-induced transformations evolving through a chemical process (hydrogen atom abstraction or elimination) following an electron transfer reaction (EC mechanism,[2] Scheme S1). On the basis of such a mechanism, simulations of the scan rate-dependence of the cyclic voltammograms were performed with the Digisim® software to estimate the rate constants of the hydrogen atom capture and release reactions at room temperature, which are given in Scheme S1. According to the well-known behavior of other electrochemically-generated radical species in acetonitrile,[3] those reactions should proceed via H-atom abstraction from the solvent and formation of molecular hydrogen, respectively.

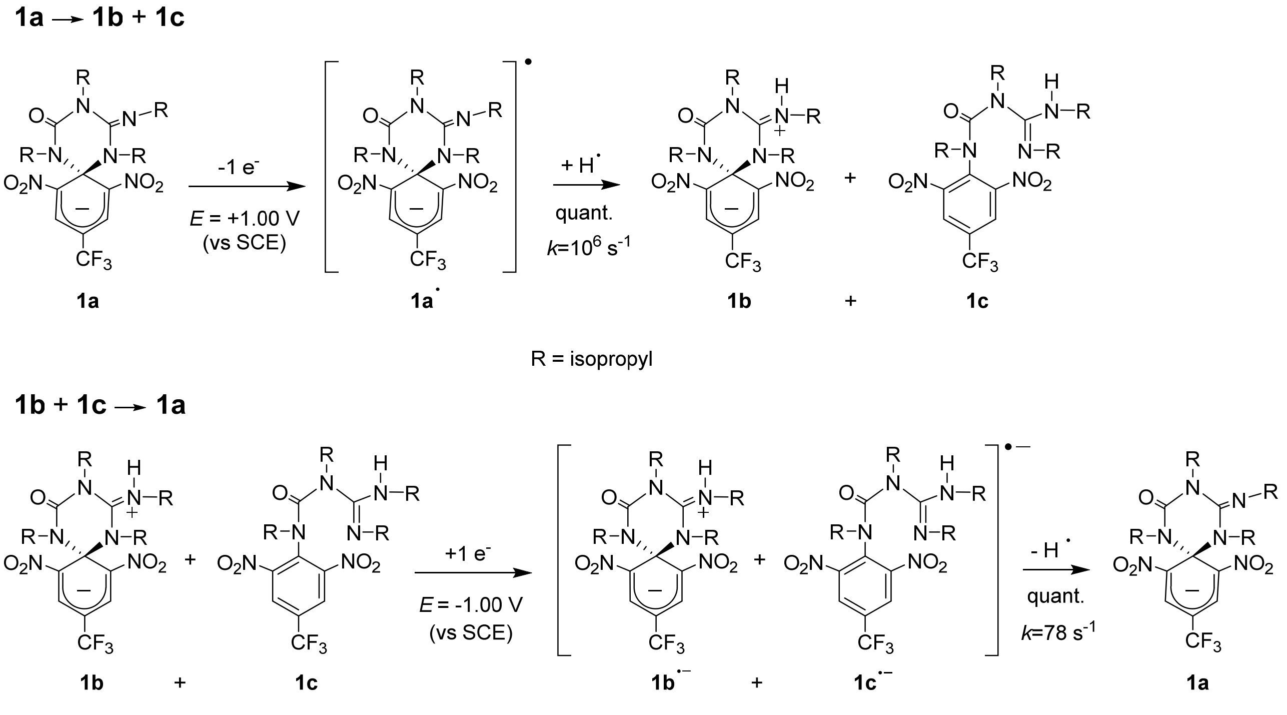

Scheme S1. Mechanism of the redox interconversion of **1** in acetonitrile. When anionic **1a** is oxidized at E = +1.00 V (vs SCE), its neutral radical species **1a$^{\bullet}$** is formed, which abstracts a hydrogen atom from the solvent to produce **1b** and, concomitantly, its tautomer **1c**. Since the

mixture **1b** + **1c** is oxidized at higher potentials (E^0 = +1.37 V (vs SCE)), these species are directly generated in their neutral zwitterionic state. On the other hand, when **1b** and **1c** are reduced at *E* = -1.00 V (vs SCE), their radical anionic forms are produced, which convert into **1a** upon elimination of a hydrogen atom. Since **1a** reduces at more negative potentials (E^0 = -1.14 V (vs SCE)), it is generated in its anionic state.

Acid-base switching:

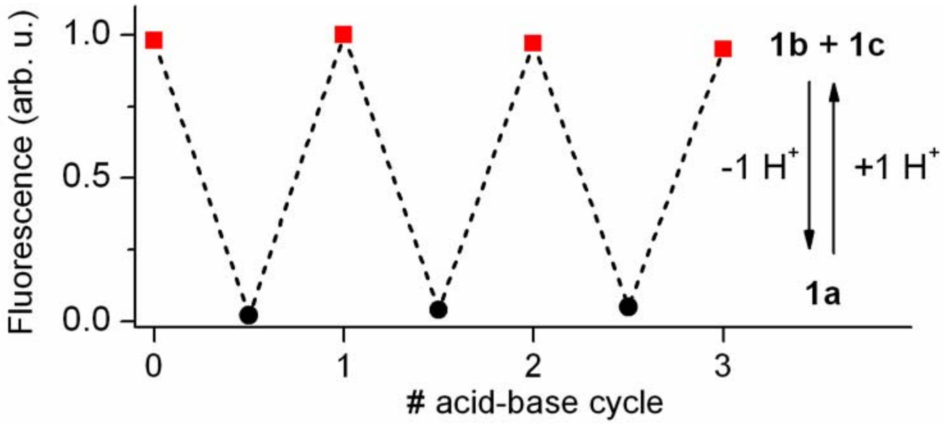

Fig. S1. Fluorescence intensity of **1** in acetonitrile at 298 K (5 10^{-5} M) upon consecutive addition of concentrated acetonitrile solutions of *n*-Bu_4NOH and $HClO_4$ to induce reversible switching between **1b+1c** and **1a** via deprotonation and protonation processes, respectively. In each cycle, acid (or base) were slowly added until no additional changes were observed by means of UV-vis absorption spectroscopy, thus indicating full conversion between the protonation states of **1**.

Temperature switching: The thermally controlled interconversion between the two isomers **1b** and **1c** of the neutral state of the switch was investigated by means of temperature dependent ^{1}H NMR experiments (from 238 to 328 K) and absorption and fluorescence measurements (from 248 K to 328 K). Separated sets of NMR signals were detected for **1b** and **1c**, whose relative intensity varied with temperature (Fig. S2). This indicated that the equilibrium constant of the tautomerization process interconverting both isomers ($K_{eq,\mathbf{1b\text{-}1c}}$) thermally shifts, the spyrocyclic compound **1b** becoming more stable upon cooling. This is clearly demonstrated in Table S1, where we show the $K_{eq,\mathbf{1b\text{-}1c}}$ values determined from the integrals of the cyclohexadiene (**1b**, δ~ 8.19 ppm) and aromatic (**1c**, δ~ 8.50-8.60 ppm) ^{1}H

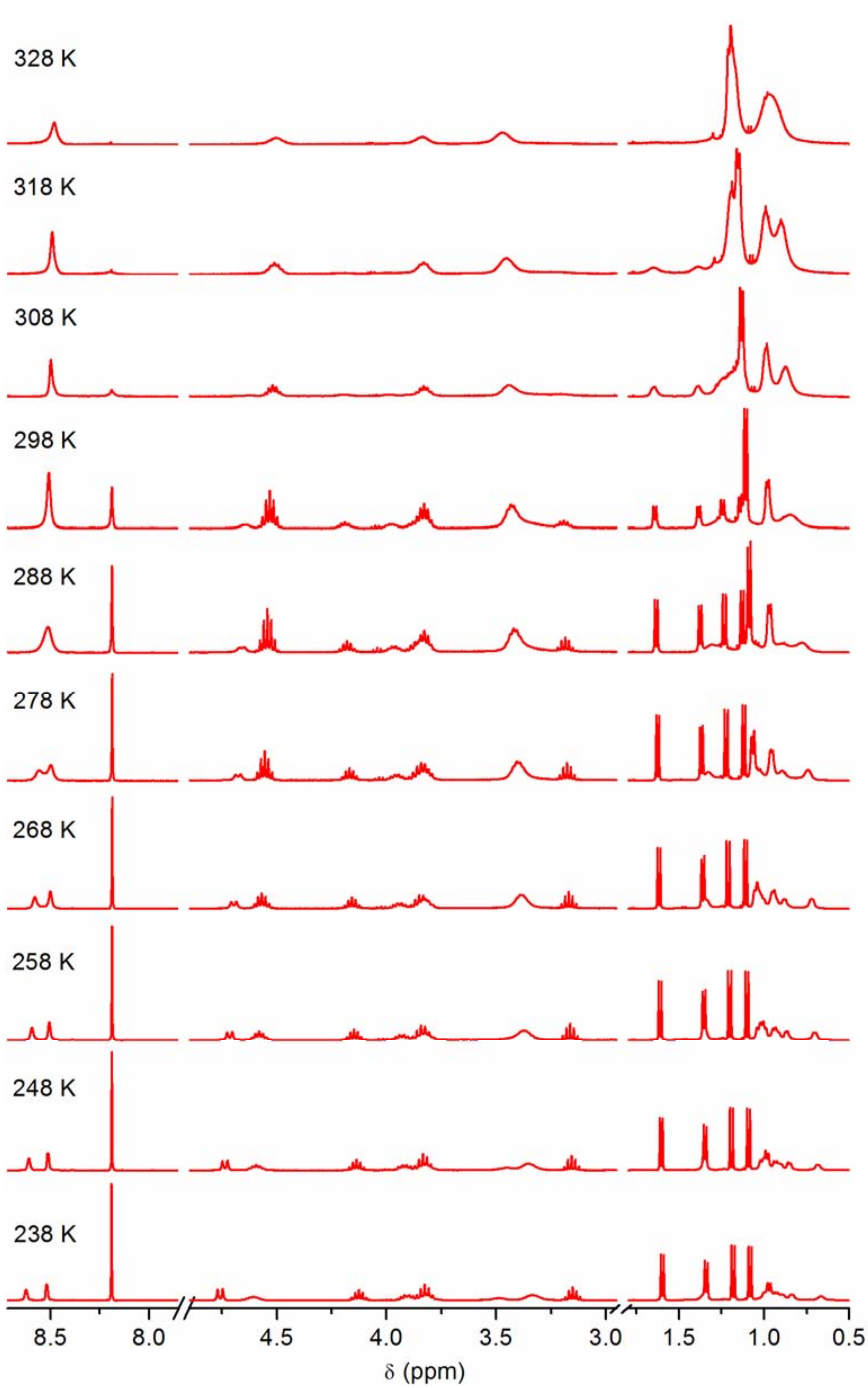

Fig. S2. ^{1}H NMR (CD_3CN, 400 MHz) spectra of the equilibrium mixture of **1b** and **1c** at different temperatures. For the sake of clarity, the intensity of the signals at $\delta > 3.0$ ppm has been magnified (x3).

NMR signals at each temperature. In addition, we could also estimate the temperature-dependence of the rate constant of the **1b** → **1c** tautomerization reaction ($k_{1b\rightarrow1c}$) by further analyzing the cyclohexadiene ^{1}H NMR signal of **1b**. The linewidth of this signal remained unaltered from 238-278 K, thus indicating the absence of dynamic effects and $k_{1b\rightarrow1c}$ values being smaller than 1 s^{-1}.[4] At higher temperatures, however, a clear broadening of this ^{1}H NMR signal was observed, which arose from faster **1b-1c** interconversion. By simulating the lineshape of the signal at these temperatures with the WinDNMR software[5] and a kinetic model that accounts for the dynamic effects of the **1b-1c** tautomerization process,[4] the corresponding $k_{1b\rightarrow1c}$ values could be estimated (Table S1). Finally, the rate constants for the **1c** → **1b** back-reaction ($k_{1c\rightarrow1b}$) were determined from the previously calculated $K_{eq,1b\text{-}1c}$ and $k_{1b\rightarrow1c}$ values.

Table S1. Temperature-dependent equilibrium and rate constants determined for the **1b-1c** tautomerization process in CD_3CN from the ^{1}H NMR data.

**T** (K)	$K_{eq,1b\text{-}1c}$	$k_{1b\rightarrow1c}$ (s^{-1})	$k_{1c\rightarrow1b}$ (s^{-1})
238	0.901	n.m.[a]	n.m.[a]
248	1.04	n.m.[a]	n.m.[a]
258	1.30	n.m.[a]	n.m.[a]
268	1.60	n.m.[a]	n.m.[a]
278	2.01	n.m.[a]	n.m.[a]
288	2.51	1.16	0.462
298	3.22	4.88	1.52
308	4.15	24.2	5.82
318	6.35	64.9	10.2
328	14.9	196	13.2

[a] Nonmeasurable since no dynamic effects were observed for the ^{1}H NMR signal of the cyclohexadiene protons of **1b**, which preserved the same linewidth over the 238-278 K range.

After a complete heating-cooling cycle, no thermal degradation of the **1b**+**1c** mixture was observed and the ^{1}H NMR spectrum then measured at room temperature matched that initially registered (Fig. S3). Similarly, changes in absorption (Fig. S4) and fluorescence (Fig.

5c) were also observed with temperature owing to the interconversion between visible-absorbing and -emitting **1b** and optically-inactive **1c**.

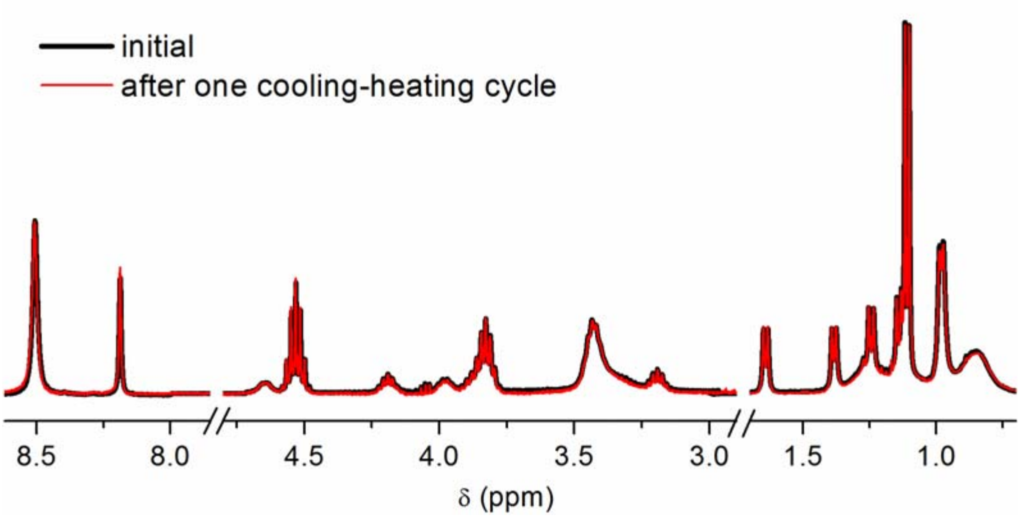

Fig. S3. ^{1}H NMR (CD_3CN, 400 MHz) spectra of the **1b**+**1c** equilibrium mixture at 298 K before and after performing the temperature dependent experiment shown in Fig. S2, where the sample was cooled down to 238 K and heated up to 328 K before recovering room temperature. For the sake of clarity, the signals at δ > 3.0 ppm have been magnified (x3).

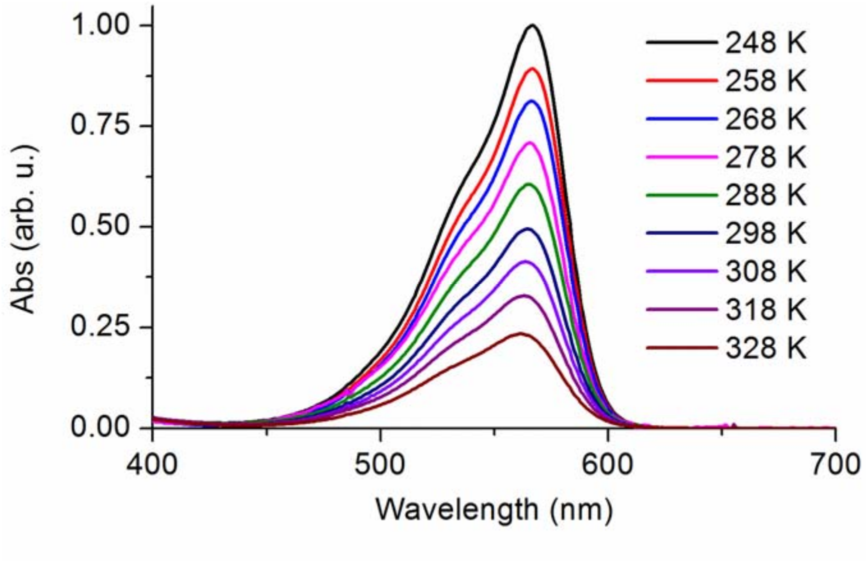

Fig. S4. Variation of the absorption spectrum of a mixture of **1b** and **1c** in acetonitrile with temperature.

V. BIBLIOGRAPHY

1 Y. Zhang, Z. Xu, L. Cai, G. Lai, H. Qiu, Y. Shen, *J. Photochem. Photobiol. A* 2008, **200**, 334.

2 I. Gallardo, G. Guirado, *Electrochem. Commun.* 2007, **9**, 173.

3 (*a*) F. M'Halla, J. Pinson, J. M. Savéant, *J. Am. Chem. Soc.* 1980, **102**, 4120; (*b*) C.P. Andrieux, J. Pinson, *J. Am. Chem. Soc.* 2003, **125,** 14801; (*c*) A. T. Krishnan, S. Chakravarthi, P. Nicollian, V. Reddy, S. Krishnan, *Appl. Phys. Lett.*, 2006, **88**, 153518.

4 (*a*) A. Steigel in *NMR Basic Principles and Progress: Dynamic NMR Spectroscopy* (Ed.: P. Diehl, E. Fluck, R. Kosfeld), Springer-Verlag, Berlin, 1978; (*b*) A. D. Bain, G. J. Duns in *Methods for Structure Elucidation by High-Resolution NMR* (Ed.: G. Batta, K. E. Kövér, C. Szántay, Jr.), Elsevier, Amsterdam, 1997.

5 H. J. Reich, *J. Chem. Educ.* 1995, **72**, 1086.
